# Disease Progression and Obstetric Outcomes of Women with Multiple Sclerosis at a Reference Center in Northeastern Brazil

**DOI:** 10.1055/s-0040-1722157

**Published:** 2021-04-15

**Authors:** Gabrielle Maria Carvalho de Barros, Bianca Etelvina Santos de Oliveira, Gabriela Januário Oliveira, Rômulo Kunrath Pinto Silva, Thiago Nóbrega Cardoso, Sabina Bastos Maia

**Affiliations:** 1 Department of Obstetrics and Gynecology, Centro de Ciências Médicas, Universidade Federal da Paraíba, João Pessoa, PB, Brazil; 2 Centro de Referência em Esclerose Múltipla da Paraíba, Fundação Centro Integrado de Apoio ao Portador de Deficiência, João Pessoa, PB, Brazil; 3 Departamento de Obstetrícia e Ginecologia, Faculdade de Medicina, Universidade de São Paulo, São Paulo, SP, Brazil

**Keywords:** multiple sclerosis, pregnancy, postpartum period, infant, newborn, abortion, esclerose múltipla, gravidez, período pós-parto, bebê, recém-nascido, aborto

## Abstract

**Objective** To describe the obstetric outcomes of patients with multiple sclerosis (MS) and the impact of pregnancy and the postpartum period on the progression of the disease.

**Methods** A case series study performed between December 2019 and February 2020, reporting pregnancies occurred between 1996 and 2019. The subjects included were women with MS undergoing follow-up at an MS referral center in Northeastern Brazil, and who had at least one pregnancy after the onset of MS symptoms, or who had their first relapse in the first year after delivery.

**Results** In total, 26 women and 38 pregnancies were analyzed – 32 of them resulted in delivery, and the remaining 6, in miscarriages. There was a significant increase in the prevalence of relapse during the postpartum period when compared with the gestational period. In 16 (42.1%) of the pregnancies, there was exposure to disease-modifying therapies (DMTs) – 14 (36.8%), to interferon β, and 2 (5.3%), to fingolimod. Higher rates of abortion, prematurity and low birth weight were reported in the group was exposed to DMT when compared with the one who was not.

**Conclusion** In the sample of the present study, there was a significant increase in the rate of MS relapse during the postpartum period when compared with the gestational period. Additionally, it seems that exposure to DMTs during pregnancy may affect the obstetric outcomes of the patients.

## Resumo

**Objetivo** Descrever os desfechos obstétricos de pacientes com esclerose múltipla (EM) e o impacto da gravidez e do período pós-parto na progressão da doença.

**Métodos** Uma série de casos realizada entre dezembro de 2019 e fevereiro de 2020, que retrata gestações ocorridas entre 1996 e 2019. As pacientes incluídas neste estudo foram mulheres com EM, que realizam acompanhamento em um centro de referência em EM no Nordeste do Brasil, e que tiveram ao menos uma gestação após o início dos sintomas da EM, ou tiveram o primeiro surto da doença no ano posterior ao parto.

**Resultados** No total, 26 mulheres e 38 gestações foram avaliadas – dentre as quais, 32 resultaram em partos, e 6, em abortamentos. Houve um aumento significativo na prevalência de surtos durante o pós-parto quando comparado com o período gestacional. Em 16 (42,1%) das gravidezes, houve exposição a terapias modificadoras da doença (TMDs) – 14 (36,8%) a β-interferona, e 2 (5,3%) a fingolimode. As taxas de abortamento, prematuridade e baixo peso ao nascer foram mais elevadas no grupo exposto às TMDs quando comparado com o não exposto.

**Conclusão** Na amostra deste estudo, houve um aumento significativo na taxa de surtos da EM durante o período pós-parto quando comparado com o período gestacional. Além disso, a exposição às TMDs durante a gestação pode afetar os desfechos obstétricos das pacientes.

## Introduction

Multiple sclerosis (MS) is an inflammatory chronic disease that affects the central nervous system. 1 2 Due to a complex immune response, varying degrees of demyelination, axonal loss and metabolic changes occur, often progressing to neurological disability. 2 3 The course of the disease is usually characterized by periods of acute neurological affection (MS relapses, attacks or exacerbations) interspersed with periods of stability, since the relapsing-remitting MS (RRMS) clinical course is the most common form of the disease. Despite this, other clinical courses – such as primary progressive MS (PPMS) and secondary progressive MS (SPMS) – do present themselves with continuous progression of the disability, regardless of the occurrence of relapses. 4

Multiple sclerosis mainly affects young women aged between 20 and 40 years, but it does not seem to have a negative impact on their fertility. 3 5 However, in previous decades, the little variety and effectiveness of the available disease-modifying therapies (DMTs) led many patients to disregard motherhood due to the fear of disability. Nevertheless, in the past few years there has been a substantial improvement in the development of DMTs, which brought about a better opportunity for disease control and an increase in the desire for motherhood. 6

During pregnancy, hormonal and immunological alterations promote significant changes in the behavior of MS. 7 8 The rate of relapses during pregnancy tends to decrease continuously over the three trimesters. In the postpartum period, however, the rate generally increases above prepregnancy levels, until, within a few months, it returns to regular levels. 9 10 11 12 13 14 The course of the disease does not seem to be affected in the long term. 14

Regarding the obstetric outcomes, studies 6 15 16 17 suggest that the neonates of patients with MS may have their development affected, especially if exposed to a DMT in the initial weeks of pregnancy, since some medications may impair fetal development. In Brazil, ∼ 55% of pregnancies are unplanned, so the exposure may be even higher than in other locations. 18

There aren't many publications available about the relationship between MS and pregnancy in Brazil. 14 19 Thus, the present study aims to evaluate the changes in MS during and after pregnancy, and to describe the obstetric outcomes of patients followed up at an MS reference center in Northeastern Brazil.

## Methods

The present work consists of a retrospective and descriptive case series, with cross-sectional and quantitative design. The necessary data were obtained through a review of medical records and telephone interviews with the patients. Data collection took place between December 2019 and February 2020, and the pregnancies occurred between 1996 and 2019.

The sample was composed of female patients, with confirmed diagnosis of MS, according to the revised McDonald criteria, who consult with a MS specialist at Centro de Referência de Esclerose Múltipla da Paraíba (CREMPB), located at Fundação Centro Integrado de Apoio ao Portador de Deficiência (FUNAD), in the city of João Pessoa, state of Paraíba, Brazil. 4 All patients had at least one pregnancy after the onset of symptoms, or had the first attack of the disease in the first year after delivery. The exclusion criteria were: patients under 18 years of age; patients who were still pregnant; and women who had not yet completed 1 year of postpartum. We performed convenience sampling, thus all patients who met the criteria of the research were included, since MS is still considered a rare disease.

The data collected included: the patient's age; number of pregnancies and when they happened; the number of abortions and trimester of loss; the type of delivery and type of anesthesia used; specifics regarding the newborn (weight and gestational age at birth); and the period during which exclusive and complementary breastfeeding occurred. Furthermore, worsening of the chronic MS symptoms; exacerbations of MS during pregnancy; gestational exposure to a DMT; and MS relapses that took place up to one year after delivery were also evaluated.

The definition of MS relapse used in the present study consists of the appearance or reappearance of one or more MS symptoms, associated with a deterioration of the neurological examination. The condition must last at least 24 hours in the absence of fever or infection. 4

Following the definition of the World Health Organization (WHO), we considered as breastfed the infants who solely ingested breast milk (exclusive breastfeeding), and those who had their breast milk diet supplemented with other liquids and solids, including non-human milk (complementary breastfeeding). Children who did not receive any amount of breast milk were perceived as not breastfed. 20

As for abortion, we herein define it as an expulsion or extraction of a conception product without signs of life with less than 20 weeks of gestation. Newborns with low birth weight are those who weighed less than 2,500 g at birth, while newborns with high birth weight are those who weighed more than 4,000 g at birth, regardless of the gestational age. All newborns delivered before reaching 37 full weeks of gestational age were considered premature. 21

All data were analyzed using the the Statistical Package for Social Sciences (SPSS, IBM Corp., Armonk, NY, US) software, version 23. For the association between the categorical variables, we used the Fisher exact test; and for the comparison between the numerical variables, the Student *t* -test with equal variances was used. The margin of error used was 5%.

The research project was approved by the Ethics in Human Research Committee of Centro de Ciências Médicas (CCM) at Universidade Federal da Paraíba (UFPB) (under CAAE: 24244819.3.0000.8069, opinion 3.718.929), and it respects the ethical principles set forth in resolution 466/2012 of the Brazilian National Health Council (Conselho Nacional de Saúde, CNS, in Portuguese), which is part of the Ministry of Health.

## Results

**Fig. 1:**
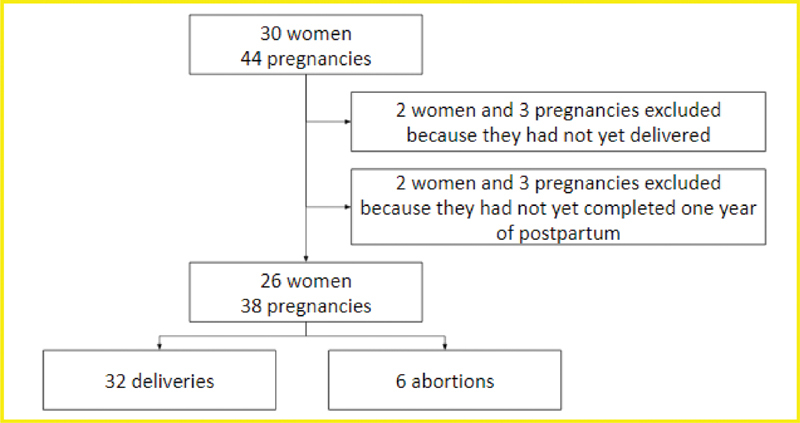
Consolidated Standards of Reporting Trails (CONSORT) flowchart of the study sample.

Overall, 30 women and 44 pregnancies were initially recruited. However, four women and six pregnancies were later excluded, since they did not meet the established criteria. Thus, 26 women and 38 pregnancies – 32 (84.2%) resulting in delivery and 6 (15.8%), in miscarriages – were finally included ( Fig. 1 ).

The median age at the onset of symptoms was 24.5 years, and the median age at diagnosis was 27 years. Most women (17; 654%), had only 1 pregnancy assessed in the present study; 8 (30.8%) had 2 gestations included; and 1 (3,.8%) had 5 pregnancies analyzed. In total, 24 (92.3%) patients had RRMS, whereas 2 (7.7%) had SPMS; 6 (23.1%) of the assessed women had their first MS relapse postpartum ( Table 1 ).

**Table 1:** General data regarding the 26 patients and 38 pregnancies

	Median (P25; P75)	n(%)
Age (years) at the onset of symptoms	24.5 (20.5; 28.75)	
Age (years) at diagnosis	27 (23.25; 29)	
Number of pregnancies assessed per patient		
*1 pregnancy*		17(65.4)
*2 pregnancies*		8(30.8)
*5 pregnancies*		1(3.8)
Clinical course of the multiple sclerosis		
*Relapsing-remitting multiple sclerosis*		24(92.3)
*Secondary progressive multiple sclerosis*		2(7.7)
Women whose first exacerbation of multiple sclerosis happened during the first year postpartum		6(23.1)
Gestational exposure to disease-modifying therapies		16(42.1)
*Interferon β*		14(36.8)
*Fingolimod*		2(5.3)
Birth weight (grams)	3,160 (2,912.5; 3,412.5)	
*Low birth weight*		3(9.4)
*High birth weight*		0(0)
Prematurity		6(18.8)
Period of exclusive breastfeeding (days)	75 (5.25; 150)	
Breastfed infants		27(84.4)
Type of delivery		
*Cesarean section*		29(90.6)
*Vaginal*		3(9.4)

Only 1 (2.6%) of the assessed pregnancies used artificial reproduction methods. The patient was using hormonal therapy, in preparation for in vitro fertilization, when she became pregnant naturally. Such pregnancy lasted until due date and there were no relapses during the gestation. However, an exacerbation happened four months after delivery.

Out of the 6 abortions – all reported by different patients – 5 (83.3%) happened during the first trimester, and only 1 (16.7%) took place during the second trimester. In 3 (50%) of these gestations, there was exposure to a DMT (interferon β-1a – 2 at a dosage of 44 µg and 1 at a dosage of 30 µg). When assessing the 38 pregnancies, the percentage of miscarriages among women who were undergoing a DMT at conception was 18.8% (3 out of 16) and 13,6% (3 out of 22) amongst those who were not; however, *p* = 0.682.

Considering only the pregnancies that resulted in delivery, in 13 (40.6%) of them there was exposure to a DMT – 11 (34.4%) to interferon β, and 2 (6.3%) to fingolimod. As shown in Table 2 , gestational relapses occurred in 5 of these 32 pregnancies (15.6%), all in different patients – 1 (3.1%) during the first trimester; 2 (6.3%) in the second; and 2 (6.3%) during the third. In the first year following delivery, 19 relapses took place after 18 (56.3%) of these births: 1 woman presented with 2 episodes of MS exacerbation in that period. In all, 21 of the 32 pregnancies (65.6%) had a related episode of exacerbation, either during the gestation itself or during the postpartum period. Out of the relapses that occurred in the first year postpartum, 11 (57.9%) happened up to 90 days after the birth; 4 (21.1%), between 90 and 180 days after the delivery; and 4 (21.1%), between 180 and 360 days.

**Table 2:** Multiple sclerosis relapses during pregnancy *versus* during the postpartum period

Relapse(s)	Pregnancy n(%)	Relapse(s)	Postpartum n(%)
Yes	5(15.6)	Yes	18(56.3)*
1st trimester	1(3.1)	0 to 90 days	11 (57.9)
2nd trimester	2(6.3)	90 to 180 days	4 (21.1)
3rd trimester	2(6.3)	180 to 360 days	4 (21.1)
No	27(84.4)	No	14(43.8)
Total	32 (100%)	Total	32 (100)

Note: *One woman had two relapses in the year after delivery.

In 29 (90.6%) pregnancies, the chosen type of delivery was cesarean section, while the remaining 3 (9.4%) were delivered via vaginal birth. Spinal anesthesia was used in 23 (71.9%) deliveries; epidural was used in other 6 (18.8%) women; 2 (6.3%) women did not receive any kind of anesthesia; and 1 (3.1%) woman could not recall which kind of anesthesia had been used. There were no statistically significant associations between the occurrence of relapses during the postpartum period and the different types of delivery ( *p* = 1.000) and anesthesia ( *p* = 0.480).

Altogether, 27 (84.4%) infants were breastfed, with 24 (75%) having undergone some period of exclusive breastfeeding. When comparing the group of patients who presented postpartum relapses with the group who did not, the median of days spent on exclusive breastfeeding was higher in the group with no exacerbations; however, *p* = 0.612.

When assessing the relationship between gestational exposure to a DMT and the outcomes of low birth weight and prematurity, the group of patients who were exposed had higher percentages for both events – 2 (15.4%) versus 1 (5.3%) regarding low birth weight ( *p* = 0.552); and 3 (23.1%) versus 3 (15.8%) for prematurity ( *p* = 0.666); however, *p* > 0.05. There were no reports of neonatal deaths or birth defects ( Table 3 ).

**Table 3:** Data on the possible effects of disease-modifying therapies on obstetric outcomes

	Exposed to disease-modifying therapies n = 13 n(%)	Not exposed to disease-modifying therapies n = 19 n(%)	*p* -value
Low birth weight	2(15.4)	1(5.3)	*p* = 0.552*
Prematurity	3(23.1)	3(15.8)	*p* = 0.666*
Neonatal death	0		
Birth defects	0		

Note: * According to the Student *t* -test with equal variances.

## Discussion

Overall, there was a significant increase in the rate of postpartum MS relapses when compared with the gestational period. Artificial reproductive techniques were only used in 1 (2.6%) pregnancy. Exposure to a DMT occurred in 16 (42.1%) pregnancies, including 2 accidental exposures to fingolimod. A high rate (90.6% [29]%) of cesarean sections was reported. The group of patients who did not have postpartum MS relapses presented a higher median of exclusive breastfeeding days when compared with the group that had postpartum relapses.

Despite current scientific evidence that MS has no significant influence on fertility, it is possible for some patients to present an association of MS and infertility, which may lead some couples to resort to artificial reproduction methods. However, studies show that the rate of relapse increases after using such techniques. Said increment is possibly due to the association of different factors: the suspension of the DMT; the stress associated with the process; and immunological changes induced by hormone therapy. 5 22 23 24

The only gestation evaluated in the present study that happened with the help of some kind of reproduction technique turned out to result in a relapse four months after the delivery – although no exacerbations occurred during pregnancy. Still, we cannot undoubtedly connect these events, as the hormonal therapy was not followed through, and the relapse happened after the birth – a period of predisposition for this occurrence.

During pregnancy, there is an increase in the humoral immune response, which, when associated to the change in the immunological pattern of reaction of Th1 to Th2 and hormonal alterations, may promote significant changes in the clinical behavior of MS. 6 7 8 Thus, the rate of relapse continuously decreases during the three gestational trimesters, and especially during the last one, in which the rate reaches its lowest numbers. After delivery, however, there is an important increase in said rate, which then declines and returns to its prepregnancy levels within four to six months. 9 10 11 12 13 14

The sample assessed in the present research behaved similarly to what has just been described, since there was a remarkable difference between the prevalence of relapse during pregnancy and in the year following delivery. Despite this, two patients reported an exacerbation during their third trimester, which is rather unusual. However, one of these women, who had both sensory and motor symptoms in the left lower limb, did present with a Zika virus infection just days before the relapse. There was no evidence of congenital syndrome related to the viral infection, but the patient went into premature labor at 34 weeks of gestational age. There were no postpartum exacerbations. The gestational relapse was probably due to the virus, since other infections have been identified as triggers for MS activity. 25 As to the Zika virus itself, so far there are few studies linking it to MS, but some reports 26 27 suggest that it might induce deterioration of the neurological condition.

Among the analyzed pregnancies, exposure to DMTs reached 42.1% of the sample, which is similar to the rate observed in other studies. 17 28 All patients discontinued such therapies after the pregnancy was diagnosed. The outcomes of abortion, low birth weight and prematurity were more frequent in the group of exposed patients; however, *p* > 0.05. In total, 2 (5.3%) pregnancies developed with exposure to fingolimod, which is in fact rare, since there currently are ∼ 500 reports of said event in the literature. 6 Both pregnancies were successful, with no gestational exacerbations. Neither of the newborns was premature, nor did any present with birth weight alterations. However, both women reported a relapse in the first 90 days postpartum.

Currently, there are no class-A DMTs for use during pregnancy according to the Food and Drug Administration. 6 29 However, when it comes to older medications such as interferon β, it is recommended to interrupt treatment right when the pregnancy is diagnosed, in light of a tendency this medication to increase the rate of prematurity. 6 Nevertheless, some specialists still consider maintaining its use during pregnancy in selected cases. 6 29 As for fingolimod, a washout period of at least two to three months is recommended, since some studies suggest 30 31 that it might be associated with fetal malformations. Despite this, there is a considerable risk of disease reactivation due to sudden DMT withdrawal. 6 In the present study, there were no cases of malformations among the exposed fetuses, and there is a possibility that the postpartum relapses may have occurred due to a natural tendency toward exacerbation often verified during that period. Still, further studies are necessary to assess the effects of the exposure to fingolimod during pregnancy, as well as the consequences of its suspension.

In regards to prenatal care, pregnancies in women with MS are not, at first sight, considered high-risk – unless there is an important disability status or other comorbidities. When it comes to the type of delivery, it follows an obstetric indication. 6 29 Although MS has been recognized as a risk factor for cesarean section, because of fatigue, spasticity of the lower limbs, slower progression of labor and/or pelvic dysfunction (possible features of MS), it has been suggested that cultural characteristics also play a role in this context. 6 32 The rate of cesarean sections in our sample was considered overly elevated (90.6%), since most studies present rates around 40% or lower. 9 10 13 32 This probably happened due to the disability caused by MS, but also due to a Brazilian cultural tendency to opt for surgical births – the national rate was around 56% in 2018–, in addition to possible fear among both obstetricians and neurologists of putting these women through the stress of vaginal birth. 33 Therefore, it is important to restate that, after an obstetric evaluation, if no deterrent factors are identified, vaginal delivery is considered safe for these patients. 6 29

Furthermore, breastfeeding has been pointed out as a possible protective factor regarding postpartum relapses, since some studies show a significant difference between the rate of relapse in women who breastfed exclusively and those who did not breastfeed or did so as part of a complementary diet. 34 35 Among the pregnancies that resulted in birth, a considerable percentage of the newborns (75%) went through some period of exclusive breastfeeding, and the group of patients who did not present postpartum relapses had a higher median of exclusive breastfeeding days when compared with the group with relapses, but *p* > 0.05. This discussion still needs to be clarified, since it is difficult to establish whether women who breastfeed for a longer period have fewer postpartum relapses or if they already have a lower relapse rate, thus enabling them to postpone the return to the use of DMT and hence making it possible for them to breastfeed for longer periods.

When it comes to the limitations of the present study, we would like to highlight its small sample, which is due to the low prevalence of MS, as it is a rare condition. The present work also has an observational and retrospective design, which makes it vulnerable to a greater number of biases when compared with other types of studies. However, this format is adequate and should be used for rare diseases such as MS.

## Conclusion

In the present study, there was a significant reduction in the rate of MS relapses in the gestational period when compared with the postpartum period. The rate of DMT exposure during conception was similar to what has been reported by other studies: most women were on interferon β, although two cases of fingolimod exposure were also identified. We encourage the performance of new studies to assess the evolution of multiple sclerosis during the gestational and puerperal cycles, to provide more tangible scientific evidence.
